# Impact of the Metal–Organic Frameworks Polymorphism on the Electrocatalytic Properties of CeO_2_ toward Oxygen Evolution

**DOI:** 10.1021/acsomega.4c08837

**Published:** 2024-11-29

**Authors:** Nicolle Pauline de Araújo Mendes, Antonio Lopes de Souto Neto, Johnnys da Silva Hortêncio, André L. Menezes de Oliveira, Rafael A. Raimundo, Daniel Araújo Macedo, Fausthon Fred da Silva

**Affiliations:** † Departamento de Química, Universidade Federal da Paraíba (UFPB), 58.051-900 João Pessoa, Paraíba, Brazil; ‡ Núcleo de Pesquisa e Extensão LACOM, Departamento de Química, Universidade Federal da Paraíba, 52051-85 João Pessoa, Paraíba, Brazil; § TEMA—Centre for Mechanical Technology and Automation, Department of Mechanical Engineering, University of Aveiro, 3810-193 Aveiro, Portugal; ∥ Programa de Pós-Graduação em Ciência e Engenharia de Materiais—PPCEM, Universidade Federal da Paraíba (UFPB), 58.051-900 João Pessoa, Paraíba, Brazil

## Abstract

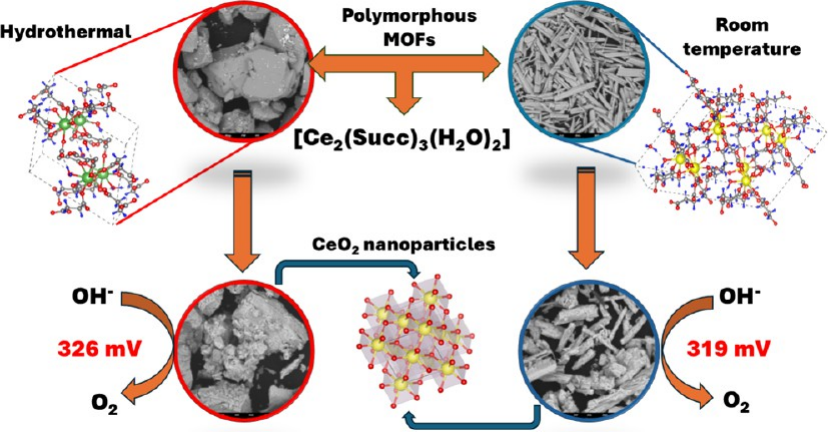

Hydrogen (H_2_) is a viable alternative as a sustainable energy source, however, new highly efficient electrocatalysts for water splitting are still a research challenge. In this context, metal–organic frameworks (MOFs)-derived nanomaterials are prominent high-performance electrocatalysts for hydrogen production, especially in the oxygen evolution reaction (OER). Here, a new synthesis of two cerium oxide (CeO_2_) electrocatalysts using Ce-succinates MOFs as templates is proposed. The cerium succinates polymorphs ([Ce_2_(Succ)_3_(H_2_O)_2_], Succ = succinate ligand) were obtained via hydrothermal reaction and room temperature crystallization, adopting monoclinic (*C*/2*c*) and triclinic (*P*1̅) crystalline structures, respectively, confirmed by X-ray diffraction (XRD). MOFs-Ce were also characterized by infrared spectroscopy (FT-IR) and scanning electron microscopy (SEM). CeO_2_ electrocatalysts were obtained via MOFs-Ce calcination at 350 °C in air, and characterized by XRD with Rietveld refinement, HRTEM, SEM, FT-IR, and Raman spectroscopy, UV–vis spectroscopy, X-ray photoelectron spectroscopy. Electrocatalytic performances were investigated in KOH 1.0 M solution, and overpotentials were η = 326 mV (for CeO_2_ (H) from monoclinic MOF-Ce) and η = 319 mV (for CeO_2_ (RT) from the triclinic MOF-Ce) for a current density of 10 mAcm^–2^. The Tafel slope values show the adsorption of intermediate oxygenated species as the rate-determining step. The high values of double-layer capacitance, the presence of oxygen vacancies, and low charge transfer resistance agree with the high performance in OER. Additionally, the materials were stable for up to 24 h, according to chronopotentiometry results.

## 1 Introduction

The significant increase in energy demand has consequently led to the intense exploitation of fossil fuels, the main energy source used. Currently, around 80% of global energy comes from these fuels.^1−4^ However, due to climate change and atmospheric pollution; the scientific community has been looking for alternative clean and renewable energy sources.^1−4^ In this context, hydrogen (H_2_) is established as a promising alternative of sustainable energy; obtained from the water electrolysis: 2H_2_O_(l)_ → 2H_2(g)_ + O_2(g)_.^5−7^ Despite the many advantages, molecular hydrogen also presents a high energy capacity or calorific value (energy per unit of weight) equivalent to 141.9 kJ/g.^7^ Water splitting through electrolysis involves two half-reactions: the oxygen evolution reaction (OER) and the hydrogen evolution reaction (HER). Since this is a nonspontaneous reaction (Δ*G*° = +237.2 kJ/mol) with high overpotential (η) mainly due to the slow kinetics of OER, which makes its practical application significantly complex for producing H_2_ to meet global demand.^8^ From this perspective, high-performance electrocatalysts were necessary for the higher technological viability of this reaction. Earth-abundant-based nanomaterials are the main focus of the state-of-the-art new high-efficiency electrocatalysts.^9,10^

Although cobalt, nickel, and iron are the majority targets in the main literature,^11,12^ cerium oxide (CeO_2_) and Ce-based electrocatalysts have recently emerged with particular interest due to their unique properties due to the presence of the Ce^3+^/Ce^4+^ redox couple.^13−17^ Cerium is the most abundant lanthanide element, even more than some d-block metals such as nickel.^13^ CeO_2_ has a fluorite crystalline structure, and it can easily shift between Ce^3+^ and Ce^4+^, leading to oxygen vacancy (V_o_) defects in response to the charge compensation mechanisms. Thus, these excellent redox properties and active oxygen vacancies make CeO_2_ extremely attractive as an electrocatalyst in OER.^13,16^ Despite these benefits, electrocatalysts based on pristine CeO_2_ still need extensive investigation, due to the low overpotential in the OER. Therefore, most investigations are focused on Ce-doped materials, or CeO_2_-based nanocomposites.^13−16^ For instance, Galani et al. synthesized CeO_2_ nanoparticles by hydrothermal synthesis to obtain a CeO_2_/RuO_2_ electrocatalyst for OER.^18^ Pristine CeO_2_ shows an overpotential of 580 mV to achieve a current density of 10 mA cm^–2^, far higher than the RuO_2_/CeO_2_ electrocatalyst (350 mV).^18^

Cerium oxide can be easily obtained using traditional methods such as coprecipitation, hydrothermal, microemulsion, and sol–gel.^14,16^ Recently, metal–organic frameworks (MOFs) were also used as templates to obtain Ce-based electrocatalysts for OER.^14,16^ MOFs are crystalline solids made up of organic ligands coordinated to metal cations, resulting in 3D-nanostructured porous structures, with high surface area.^19^ The calcination of MOFs in atmospheric air leads to the formation of uniformly dispersed metal-oxide nanoparticles, with a unique hierarchical structure that can retain the morphology and porosity of the precursor material.^20^ E.g., Nazar et al. showed the OER electrocatalytic performance of CeO_2_/C nanorod arrays, obtained from a MOF-Ce using the 1,3,5-benzene tricarboxylic acid as a ligand.^21^ The material shows an overpotential of 297 mV at 10 mA cm^–2^, and a low Tafel slope (46 mV dec^–1^).^21^ Souto Neto et al. also reported the MOF-templated CeO_2_/Co_3_O_4_ nanocomposites synthesized from ZIF-67 and cerium-succinate.^22^ In this case, the electrocatalyst showed an overpotential of 366 mV (at *J* = 10 mA cm^–2^) and chemical stability until 15 h, a superior performance compared to the pristine Co_3_O_4_.^22^

Based on all this evidence, this work evaluates the electrocatalytic performance in OER of two CeO_2_ samples (named CeO_2_ (H) and CeO_2_ (RT)), prepared using two MOF polymorphs prepared under different conditions. The two crystalline polymorphs of MOF structure have the chemical formula [Ce_2_(Succ)_3_(H_2_O)_2_] (Succ = succinate ligand) and were synthesized by hydrothermal method (MOF-Ce (H)) and crystallization at room temperature (MOF-Ce (RT)). CeO_2_ (H) and CeO_2_ (RT) were obtained from the direct calcination of the corresponding MOFs under an air atmosphere. The electrocatalysts were characterized by X-ray diffraction (XRD) with Rietveld refinement, vibrational spectroscopy (infrared and Raman), UV–vis spectroscopy, scanning electron microscopy (SEM), high-resolution transmission electron microscopy (HRTEM), and X-ray photoelectron spectroscopy (XPS). The electrocatalytic performances were evaluated using the techniques of linear sweep voltammetry (LSV), cyclic voltammetry (CV), electrochemical impedance spectroscopy (EIS), and chronopotentiometry (CP).

## 2 Experimental Section

### 2.1 Chemicals

Succinic acid (C_4_H_6_O_4_, 99%, Sigma-Aldrich), cerium(III) nitrate hexahydrate ((Ce(NO_3_)_3_·6H_2_O), 99%, Sigma-Aldrich), sodium hydroxide (NaOH, 99%, Dinamica Química) and ethanol (C_2_H_6_O, 99%) were obtained commercially and used without previous purification.

### 2.2 Synthesis of MOF-Ce (H)

First, succinic acid (59.0 mg, 0.5 mmol) was dissolved in a beaker with 10 mL of deionized water and the pH was adjusted to 5.0 using a NaOH aqueous solution (2 M). Then, this solution was transferred to a Teflon-lined hydrothermal reactor (25 mL) with cerium nitrate hexahydrate (217 mg, 0.5 mmol). The system was placed in a muffle and heated at 120 °C for 4 days and then cooled to room temperature. Resulting white crystals were rinsed with a water–ethanol solution (50% v/v), and air-dried.

### 2.3 Synthesis of MOF-Ce (RT)

Succinic acid (59.0 mg, 0.5 mmol) was dissolved in 10 mL of deionized water, and the pH was adjusted to 5.0 using a NaOH aqueous solution (2 M). Subsequently, the solution was transferred to a beaker containing 0.5 mmol of cerium nitrate hexahydrate (217 mg). The system was left at room temperature, and after a week, the resulting white crystals were collected, rinsed with a water–ethanol solution (50% v/v), and air-dried.

### 2.4 Synthesis of CeO_2_ (H) and CeO_2_ (RT)

**Figure 1:**
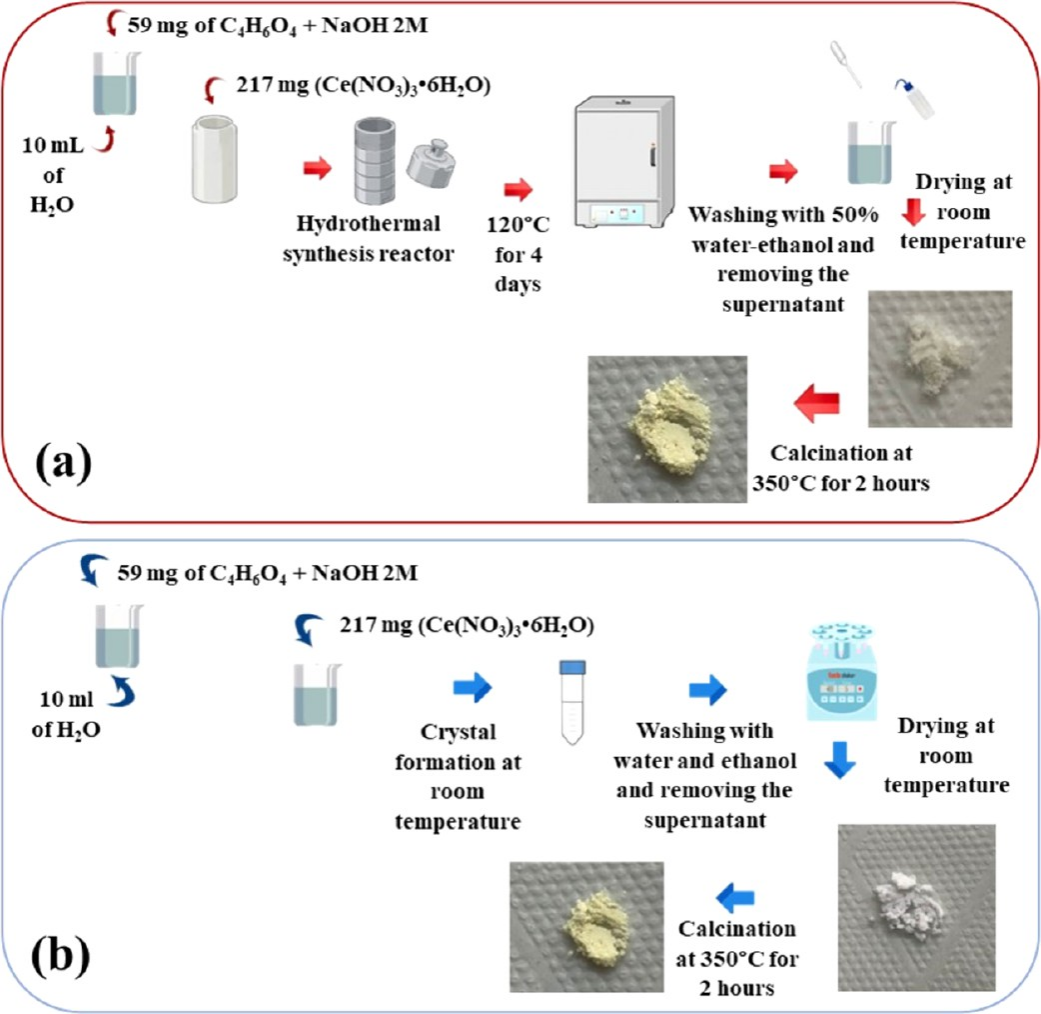
Synthetic process in (a) hydrothermal synthesis and (b) room temperature synthesis.

The MOF-Ce (H) was calcined in a porcelain crucible using a preheated muffle at 350 °C for 2 h and cooled to room temperature, to obtain the sample named CeO_2_ (H). The procedure was repeated using the MOF-Ce (RT) to obtain the sample named CeO_2_ (RT). Figure 1 shows the synthesis flowchart used to obtain MOFs and cerium oxides.

### 2.5 Chemical, Structural, and Morphological Characterization

Crystalline structures were determined via X-ray powder diffraction using a Shimadzu diffractometer, model XRD-6000, with a voltage of 30 kV, current of 30 mA with power of 2 k VA and Kα Cu radiation (λ = 1.54°). FullProf software was used for Rietveld refinement. The infrared spectra were analyzed using the Shimadzu instrument, model IR PRESTIGE-21, in a range of 4000–400 cm^–1^. Sample analyses were carried out using KBr tablets in a proportion of 1 mg of sample for 100 mg of KBr. UV–vis spectra were obtained using a Shimadzu spectrophotometer between 1100 and 190 nm, using barium sulfate as a reflectance standard. X-ray photoelectron spectroscopy was performed using an XPS spectrometer (ScientaOmicron ESCA+) with monochromatic Al Kα radiation (*h*ν = 1486.6 eV). High-resolution XPS spectra were recorded at a constant pass energy of 20 eV with 0.05 eV, and the data processing was performed using the CasaXPS software. The morphological characterizations of MOF-Ce (H) and MOF-Ce (RT) and the electrocatalysts CeO_2_ (H) and CeO_2_ (RT) were carried out using a Thermo Fisher Scientific Phenom Pro Desktop SEM scanning electron microscope and a transmission electron microscopy (TEM) in a JEOL JEM-2100 microscope.

### 2.6 Electrochemical Characterization

Electrochemical analyses were conducted using a Metrohm Autolab potentiostat/galvanostat model PGSTAT 101 using an alkaline KOH solution 1.0 M. Measurements were performed using an electrochemical cell with three electrodes: the Ag/AgCl reference electrode (KCl 3.0 M), the working electrode which is prepared using the electrocatalyst material to be analyzed (CeO_2_) and commercial nickel foam, and a platinum wire counter electrode. First, the nickel foams (with 98.8% nickel in their composition, with porosity ≥95% and a useful geometric area of 1.0 cm^2^) were treated in ultrasound for approximately 10 min using a concentrated HCl solution, followed by a solution of isopropanol or acetone and deionized water. After this treatment, the foams were dried at room temperature. Then, 5 mg of the respective electrocatalyst was suspended in a solution of 500 μL of isopropanol and 50 μL of Nafion, using ultrasonic treatment. This suspension was drop-casted on the nickel foam and dried at room temperature for 24 h. Linear sweep voltammetry (LSV) was performed to evaluate the performance of the electrocatalysts through the calculation of the overpotential (eq 1). Tafel slopes were used to evaluate the reaction kinetics (eq 2). All potentials were converted to the reversible hydrogen electrode (RHE) (eq 3).
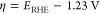
1
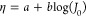
2
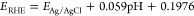
3where η is the overpotential (V), *b* is the Tafel slope (mV dec^–1^), *a* is a constant and *E*_RHE_ is the reversible hydrogen electrode potential. Cyclic voltammetry (CV) was applied to determine the double-layer capacitance (*C*_DL_) and the electrochemically active surface area (ECSA) (eq 4)
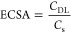
4where *C*_S_ is the specific capacitance value already established in the literature for electrocatalysts in alkaline media, whose value is 0.040 mF cm^–2^.^11^ In the electrochemical impedance spectroscopy, the polarization of the working electrodes is carried out at three potentials (1.30, 1.50, and 1.60 V), measured before, during, and after the OER. A frequency range of 0.01 Hz–10 kHz with an amplitude of 10 mV was used. EIS spectra were acquired through Z-View software using the equivalent circuit model and nonlinear least-squares fitting procedure. Chronopotentiometry (CP) was applied to evaluate the electrocatalysts’ stability. Experiments were conducted in an alkaline KOH solution of 1.0 M for 24 h.

## 3 Results and Discussion

### 3.1 MOF-Ce (H) and MOF-Ce (RT)

**Figure 2:**
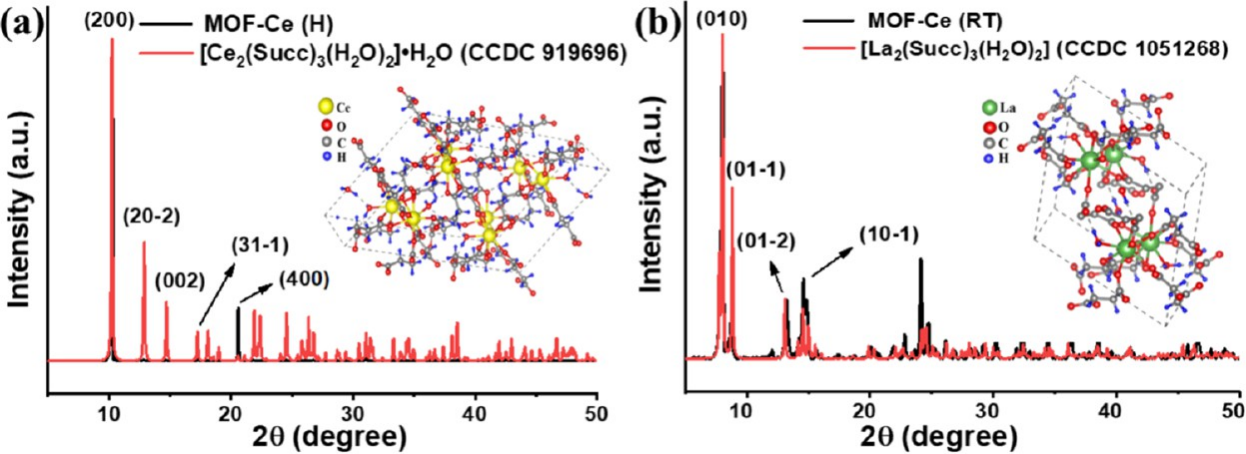
(a) XRD of MOF-Ce (H) compared to literature (CCDC 919696), (b) XRD of MOF-Ce (RT) compared to literature (CCDC 1051268).

Lanthanide succinates present a wide structural diversity, being able to form coordination polymers with a 2D or 3D structure, depending on the nature of the metal ion, synthetic method, and/or experimental parameters (pH, solvent, stoichiometric proportion, etc.).^23^ The MOFs’ crystalline structures were investigated via XRD patterns, and the results are shown in Figure 2. For the cerium succinate obtained by hydrothermal reaction (MOF-Ce(H), Figure 2a), the experimental powder pattern indicates the formation of a monoclinic structure (CCDC 919696), with *C*2/*c* space group and chemical formula [Ce_2_(Succ)_3_(H_2_O)_2_]·H_2_O (Succ = succinate), as reported by de Oliveira et al. also under hydrothermal conditions.^24^ The most intense peak refers to the (200) diffraction plane, agreeing with the literature.^24^ In this structure, cerium ions are bonded to two crystallographically independent Succ^2–^ anions (one with anticonformation and one with gauche conformation), throughout oxo-carboxyl bridge and bridge coordination modes.^24^ The Ce^3+^ coordination polyhedron is formed by eight O atoms from succinate anions and one O atom from water–water-coordinated molecule, resulting in a tricapped trigonal prismatic geometry.^24^

The diffraction pattern of the cerium succinate synthesized at room temperature (MOF-Ce(H), Figure 2b) shows a similar structure to the lanthanum succinate obtained by D’Vries and co-workers under solvothermal conditions (CCDC 1051268).^25^ In this case, the compound crystallizes in a triclinic crystal system and *P*1̅ space group, with the chemical formula [Ce_2_(Succ)_3_(H_2_O)_2_]. This lanthanide succinate also shows two crystallographically independent trivalent Ln cations, nine-coordinated by eight O atoms from succinate anions and one O atom from a water-coordinated molecule, as well.^25^ However, the coordination environment results in a trigonal prism square-face tricapped geometry. All three crystallographically independent succinate anions show oxo-carboxylate coordination mode.^25^

The infrared spectra (Figure S2) show similar signals for both MOF-Ce, related to the water molecules (hydrated and coordinated) and organic groups of the succinate ligands. Broadband between 3600 and 3200 cm^–1^ was observed, related to the presence of symmetric O–H stretching of the water molecules. Signals due to the symmetric and asymmetric stretching of the methylene groups from the succinate ligand are at 2983 and 2930 cm^–1^ for MOF-Ce (H) and 2981 and 2927 cm^–1^ for the MOF-Ce (RT), respectively. In-plane and out-of-plane C–H bending modes were observed between 1211–1170 cm^–1^. The signals at 1381, 1550, and 1580 cm^–1^ are associated with the symmetric and asymmetric stretching of the carboxyl groups coordinated with the metal cations. Additionally, low intense peaks at 900–600 cm^–1^ are related to the metal–carboxylate vibrations. All signals agree with the literature.^24,26,27^

**Figure 3:**
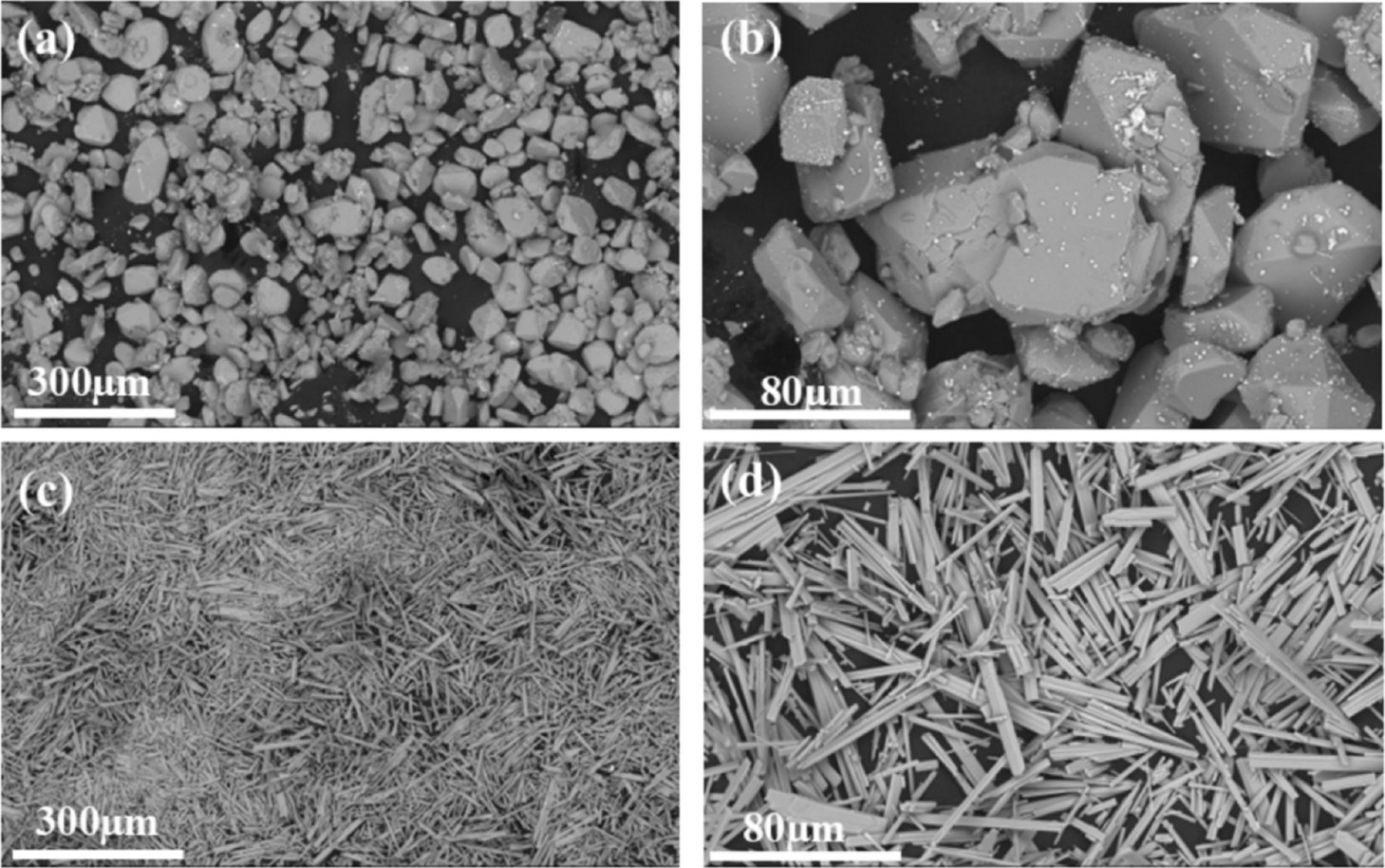
SEM images of the (a, b) MOF-Ce (H) and (c, d) MOF-Ce (RT).

Scanning electron microscopy (SEM) was used to evaluate the impact of the synthesis method on the morphology of MOF-Ce, and the images are shown in Figure 3. MOF-Ce (H) obtained via hydrothermal reaction resulted in a block-like morphology, and micrometric particles of irregular size and shape distributions (Figure 3a,b). This morphology was also obtained by Oliveira and co-workers for Tm-succinate (triclinic, *P*1̅ space group) obtained by hydrothermal method.^28^ However, for the MOF-Ce (RT) obtained in crystallization at room temperature and pressure (Figure 3c,d), rod-like morphology micrometric crystals were observed, with uniform size and shapes. Crystals with similar shape/size were also observed for Tm-succinates with a monoclinic structure^28^ and for lanthanum succinates obtained by D’Vries^25^ with a crystalline structure similar to those obtained in this work.

### 3.2 CeO_2_ (H) CeO_2_ (RT)

**Figure 4:**
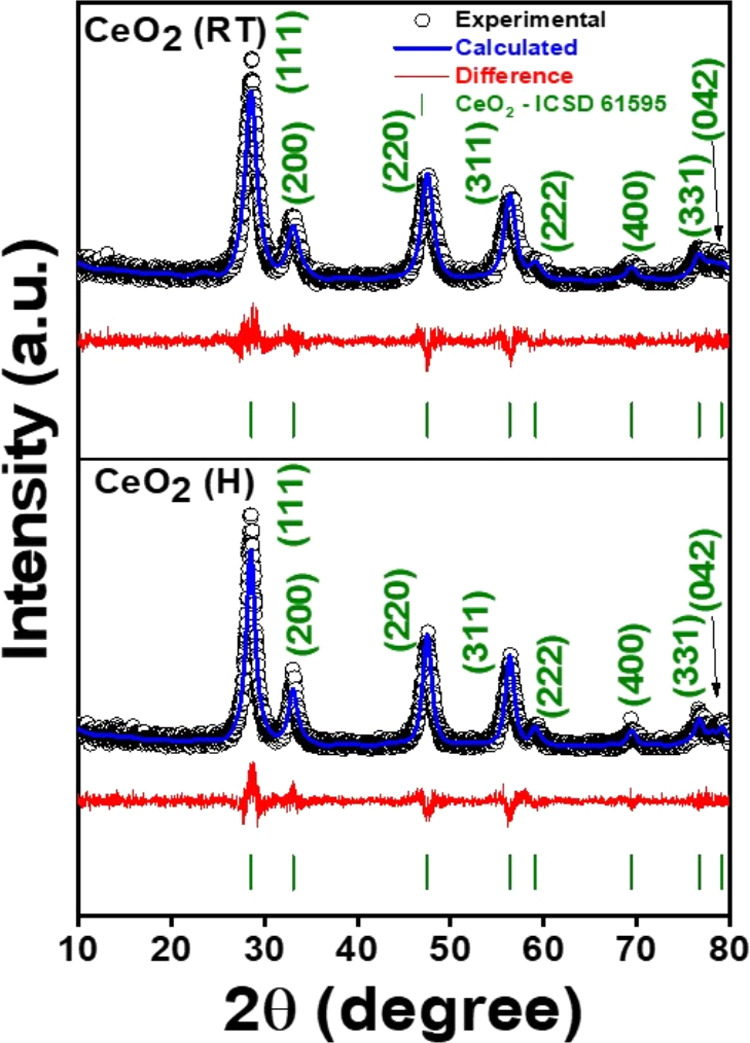
XRD patterns with Rietveld refinement of CeO_2_ (H) and CeO_2_ (RT).

Figure 4 shows the diffraction patterns of CeO_2_ (H) and CeO_2_ (RT) obtained through the calcination of MOF-Ce (H) and MOF-Ce (RT) respectively, which agree with the crystallographic reference (ICSD-61595). Cerium oxides crystallize in the fluorite crystal structure (face-centered cubic structure) with space group *Fm*3̅*m* (similar to the cubic spinel of cobaltite) in which the cations are face-centered (Ce^4+^), and the anions are located at the vertices of the cubic crystal structure.^29^ The most intense peaks near 28.4, 32.7, 47.3, and 56.51° correspond to the (111), (200), (220), and (311) diffraction planes, respectively. The Rietveld refinement data (Table S1) showed lattice parameters *a* = *b* = *c* = 5.41 Å for all samples, indicating that the precursor change did not lead to unit cell variations. Average crystallite sizes calculated were 8.3 and 6.2 nm, for CeO_2_ (H) and CeO_2_ (RT), respectively.

**Figure 5:**
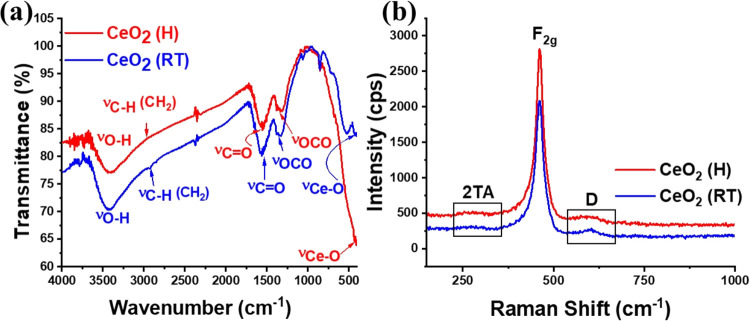
(a) FT-IR and (b) Raman vibrational spectra of CeO_2_ (H) and CeO_2_ (RT).

Figure 5a shows the FT-IR spectra for the CeO_2_ samples. The broadband between 3600 and 3200 cm^–1^ is due to O–H symmetric stretching, consistent with the presence of hydroxyl groups on the oxide surface. The signals at 2985 and 2930 cm^–1^ are related to the symmetric and asymmetric stretching of the residual methylene group. Bands at 1384 and 1340 cm^–1^; 1540 and 1580 cm^–1^ correspond to the symmetric and asymmetric stretching of the carboxyl group. All these signals are related to residual organic groups on the particle surface, due to the incomplete decomposition of the succinate ligand. Other authors also report the presence of these organic groups on the surface of CeO_2_ obtained from organic precursors.^30,31^ The vibrational frequencies were also investigated via Raman spectroscopy, and the results are shown in Figure 5b. The intense signals near 463 cm^–1^ (CeO_2_ (H)) and 460 cm^–1^ (CeO_2_ (RT)) are related to the symmetric stretching vibrational mode of the Ce–O_8_ unit in the fluorite structure, assigned as the F_2g_ phonon mode.^32^ For CeO_2_, defect-induced Raman signals can be frequently observed near 540 cm^–1^ and below 400 cm^–1^.^33^ Thus, the peaks at 273 cm^–1^ are related to the 2TA (second-order transverse acoustic) mode, due to lattice dislocated oxygen atoms. However, the signal at 590 cm^–1^ (D-band) indicates the presence of oxygen vacancy.^32,33^ The UV–vis absorption spectra (Figure S2) indicated strong absorption bands centered at 350 nm, associated with the ligand-to-metal charge transfer (LMCT) transition, from the 2p-electrons of the O^2–^ ions to the Ce^4+^ 4f-orbitals.^34^ The band gap energies (*E*_g_) were calculated using the Tauc plot method (Figure S3) and the values were 3.16 eV for CeO_2_ (H) and 3.09 eV for CeO_2_ (RT). These values are compatible with another CeO_2_ obtained through several methods in the literature.^35−38^

**Figure 6:**
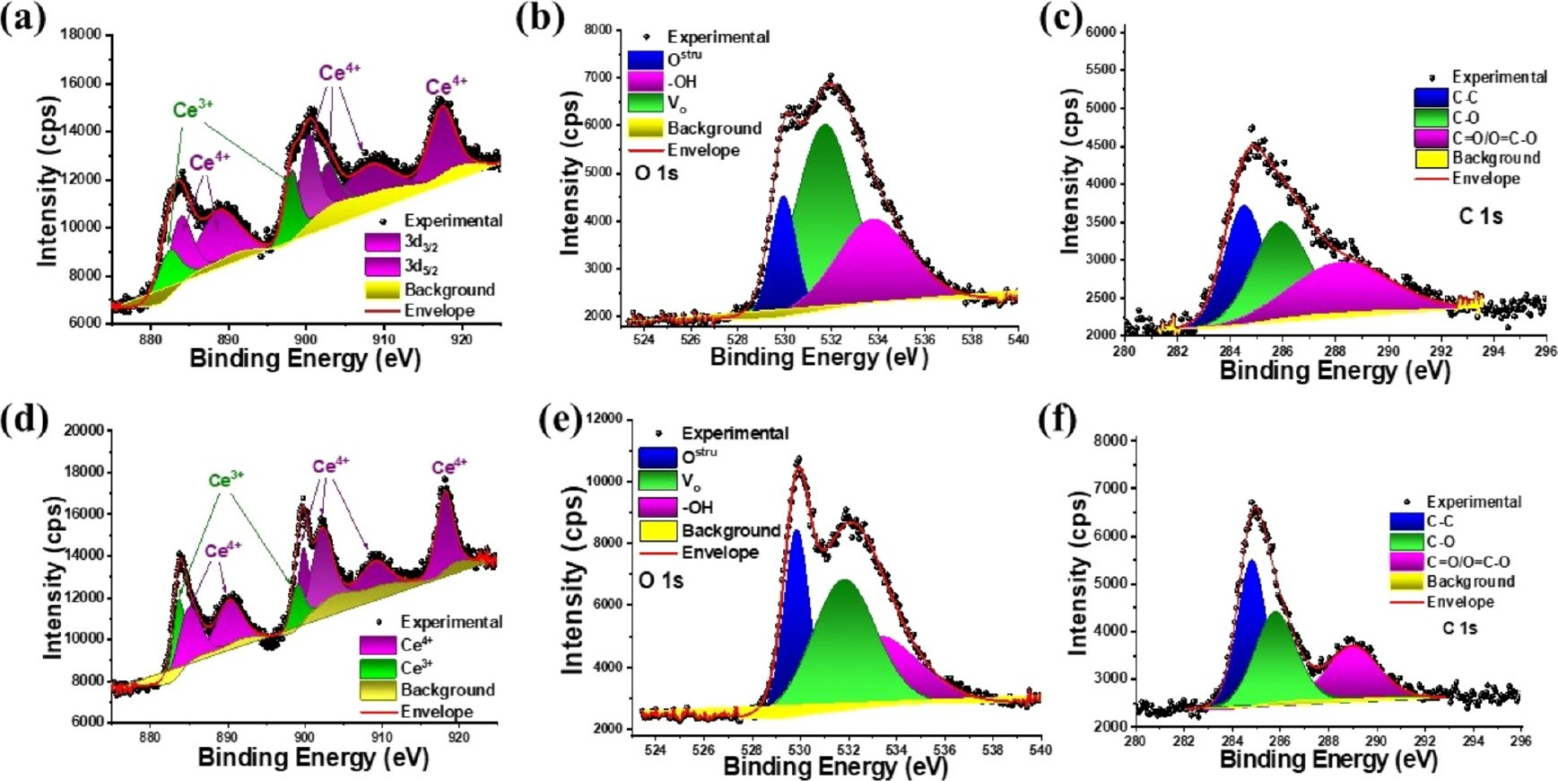
Ce 3d, O 1s, and C 1s high-resolution XPS spectra for (a–c) CeO_2_ (H) and (d–f) CeO_2_ (RT).

The surface chemical composition of cerium oxides was investigated through XPS, and results are shown in Figures S3 and 6. Survey spectra (Figure S4) show the main signals related to O 1s, C 1s, Ce 4d, Ce 3d, and Ce LMM, confirming the presence of these elements on the oxide surface. The atomic percentages on the surface (Table 1) confirm a large presence of carbon on the surface of both cerium oxides, agreeing with the FT-IR data. However, CeO_2_ (H) has a larger quantity of Ce atoms on the surface, while CeO_2_ (RT) has a higher surface oxygen content. High-resolution XPS spectra in the Ce 3d, O 1s, and C 1s emission lines are shown in Figure 6. In the Ce 3d spectra (Figure 6a,d), six well-defined peaks are expected for Ce^4+^ species, which are associated with 3d_5/2_ and 3d_3/2_ doublets. On the other hand, for the existence of Ce^3+^ cations, four peaks are expected in XPS spectra. When mixed Ce^4+^/Ce^3+^ cations coexist in a sample, a broadening of the spectrum may occur and become more complex.

**Table 1:** Atomic Percentages of Cerium, Oxygen, and Carbon Obtained by XPS Survey Spectra

	atomic percentage		
sample	cerium	oxygen	carbon
CeO_2_ (H)	24.45%	34.60%	40.95%
CeO_2_ (RT)	19.71%	37.60%	42.69%

In the present case, the high-resolution XPS spectra in the Ce 3d emission line exhibit three-lobed envelopes at around 876–896 and 895–914 eV, and up to approximately 924 eV, respectively, and the whole spectra can be deconvoluted into multiplets and correspond to spin–orbit split 3d_3/5_ and 3d_5/2_, respectively.^39,40^ The peaks were deconvoluted into Gaussian–Lorentzian components after Shirley background subtraction, listed in Table S2. The high-resolution XPS spectra in the Ce 3d emission line indicates the higher concentration of Ce as Ce^4+^ in both samples, whose components 3d_5/2_ and 3d_3/2_ are located at 884.35 and 903.18 eV for the CeO_2_ (H), and 884.97 and 902.38 eV for CeO_2_ (RT), respectively. In the case of the 3d_5/2_ and 3d_3/2_ components for Ce^3+^ species occurred at 882.77 and 898.58 eV for CeO_2_ (H), and 883.66 and 899.02 eV for CeO_2_ (RT), respectively.

The relative concentrations of Ce^3+^ and Ce^4+^ were estimated as listed in Table 2. The relative content of Ce^3+^ on the CeO_2_ (H) is 18.25%, which was higher than that observed for CeO_2_ (RT). As expected, the greater formation of Ce^3+^ species in CeO_2_ (H) is associated with the higher amount of oxygen vacancy on the surface of this material as confirmed by the deconvolution of the O 1s XPS spectra (Table 2). Although both samples present a high content of oxygen vacancies compared to structural oxygen and hydroxyl groups, CeO_2_ (H) stands out with 48.79%. The presence of Ce^3+^ associated with oxygen vacancies is commonly observed in CeO_2_ samples and has been reported by other authors,^39^ also agreeing with the Raman spectra. Deconvoluted C 1s high-resolution spectra indicate the presence of C–C bonds and oxygenated organic groups on the CeO_2_ (H) and CeO_2_ (RT) surfaces, as observed in the FT-IR data.

**Table 2:** Ce Ionic Percentage and Oxygen Concentrations Obtained from the Ce 3d and O 1s High-Resolution XPS Spectra

percentage (%)	CeO_2_ (H)	CeO_2_ (RT)
cerium		
Ce^3+^	18.25	12.45
Ce^4+^	81.75	87.55
oxygen		
*O*^estru^	14.39	24.51
*V*_o_	48.79	43.56
O–H	36.83	31.93

**Figure 7:**
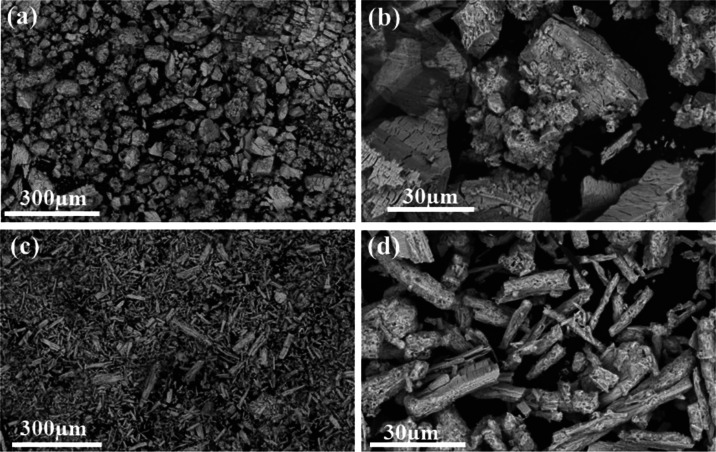
SEM images of (a, b) CeO_2_ (H) and (c, d) CeO_2_ (RT) electrocatalysts.

The morphology of cerium oxides was investigated via SEM (Figure 7), compared to the MOF precursors. In both cases, CeO_2_ nanoparticles agglomerate in the same shape as the precursor MOF morphology, showing that the cerium succinates can act as templates effectively as other MOF materials in the literature,^41−43^ thus controlling the final metal-oxide shape and size. In both cases, a reduction in cluster size was observed, in agreement with the reduced crystallite sizes obtained from the XRD data. The morphology can directly impact the cerium oxide electrocatalytic performance. E.g., Yanru and co-workers synthesized two CeO_2_, showing the effect of morphology on OER electrocatalytic activity. In this case, the CeO_2_ nanosphere obtained via hydrothermal synthesis was more effective as an electrocatalyst compared to CeO_2_ with nanowire morphology.^44^

**Figure 8:**
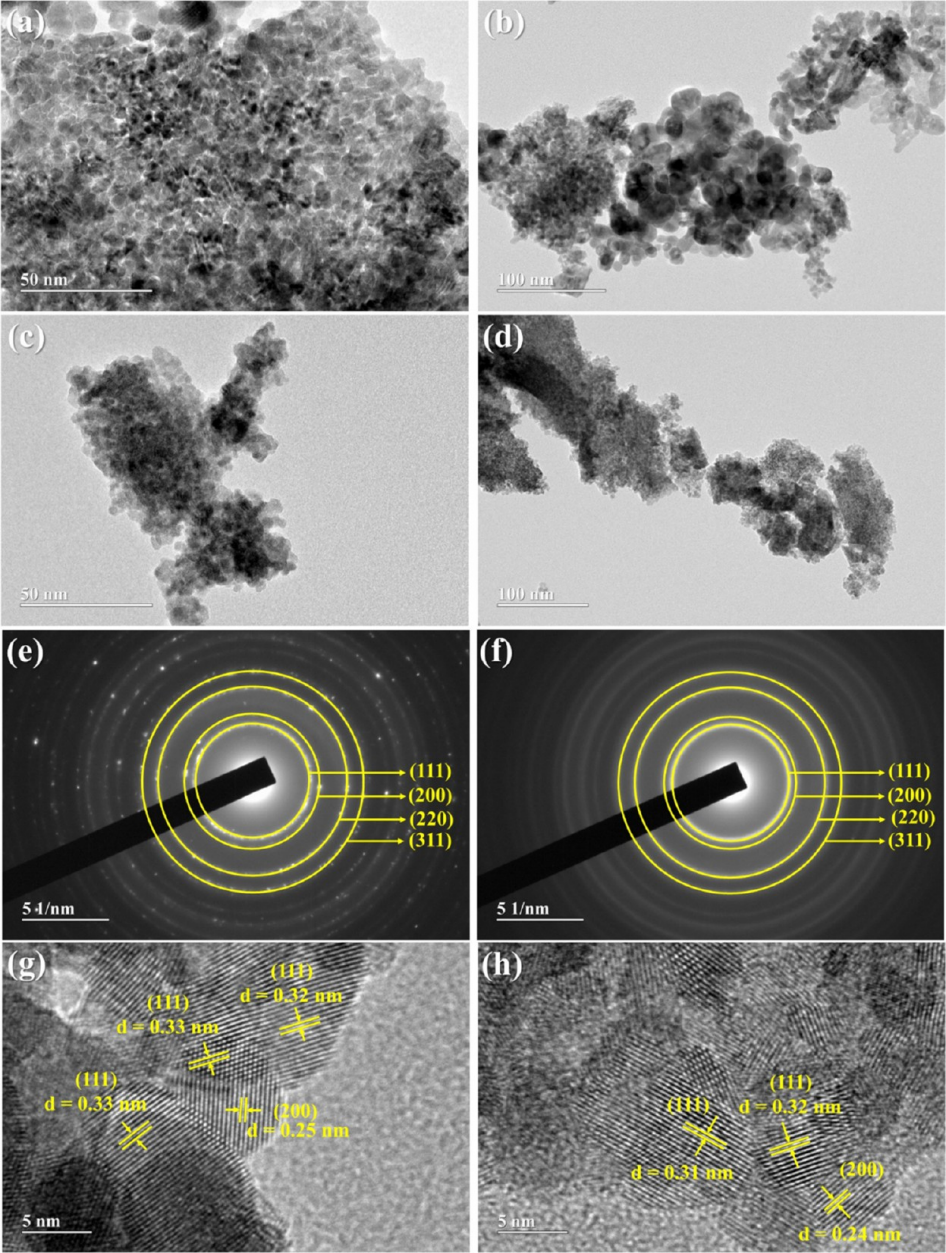
TEM images of (a, b) CeO_2_ (H) and (c, d) CeO_2_ (RT) samples, SAD analysis and high-resolution HRTEM image for the (e, g) CeO_2_ (H) and (f, h) CeO_2_ (RT), respectively.

The morphology of the CeO_2_ (H) and CeO_2_ (RT) electrocatalysts was investigated in detail using high-resolution TEM (HRTEM), and the images are shown in Figure 8. For the CeO_2_ (H) sample (Figure 8a,b), sphere-like morphology nanoparticles were observed, however, small agglomerates of larger particles were found (Figure 8b). This sample presented a very heterogeneous particle size distribution with an average size of 7.8 nm (Figure S4), in agreement with the average crystallite size obtained via XRD data. The TEM images for the CeO_2_ (RT) electrocatalyst also indicated sphere-like morphology nanoparticles, however with a homogeneous particle size distribution than the CeO_2_ (H) sample, and an average particle size equal to 4.5 nm (Figure S5), in agreement with the XRD data. Selected area diffraction (SAD, Figure 8e,f) and HRTEM images (Figure 8g,h) for both samples confirm the main diffraction planes of the fluorite cubic structure of CeO_2_.

**Figure 9:**
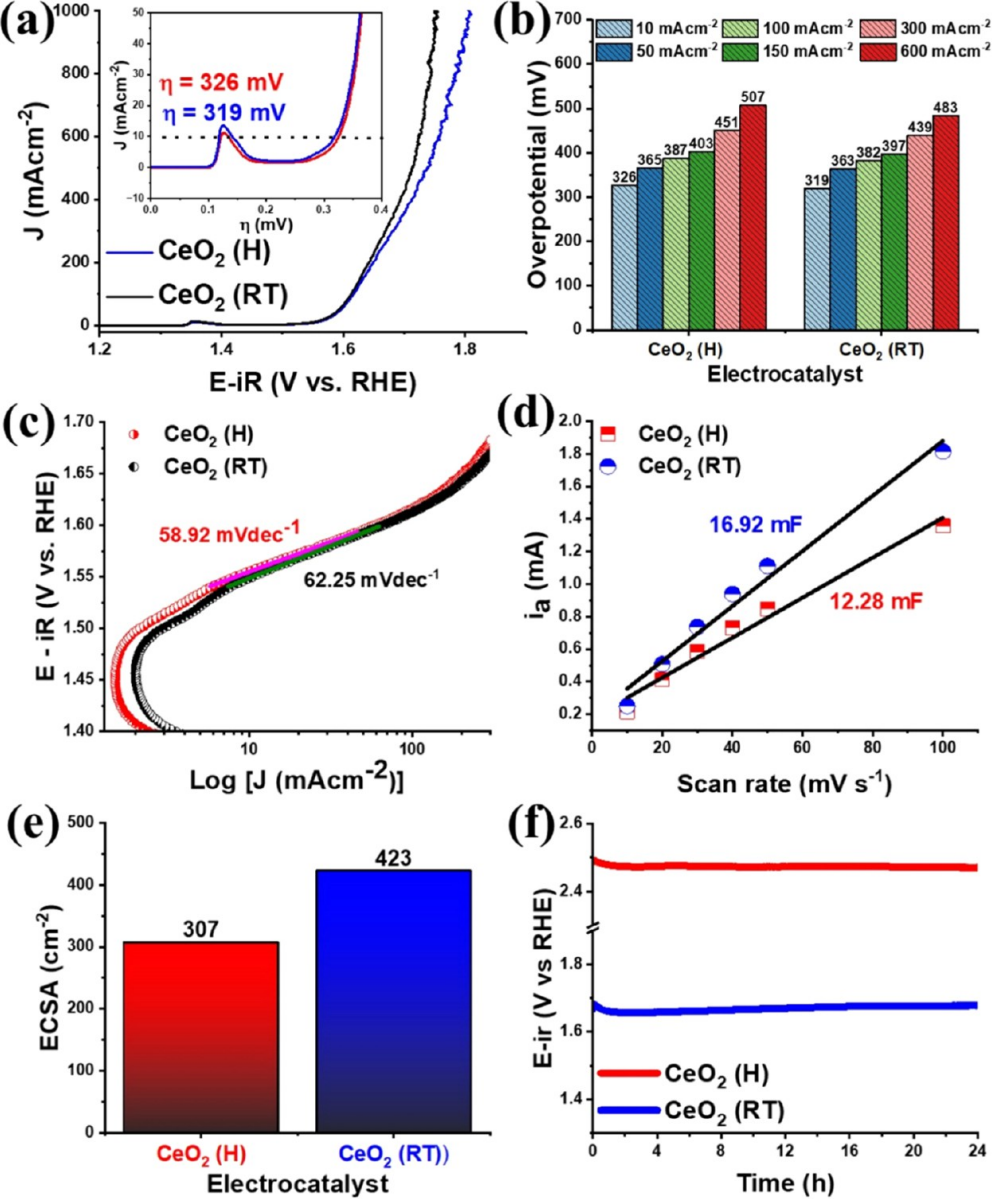
(a) LSV in alkaline KOH 1.0 M solution, (b) overpotentials at several current densities, (c) Tafel slope, (d) anodic current (*i*_a_) versus scan rate, (e) ECSA data, and (f) chronopotentiometry data for of CeO_2_ (H) and CeO_2_ (RT) electrocatalysts.

OER electrocatalytic investigations were conducted in KOH 1.0 M alkaline medium (Figure 9), and LSV measurements were first performed, resulting in overpotential values of 326 mV for CeO_2_ (H) and 319 mV for CeO_2_ (RT) vs RHE for OER to achieve *J* = 10 mA cm^–1^ (Figure 9a). These results are far superior to the pristine Ni foam substrate (516 mV, Figure S6). Thus, both electrocatalysts show high OER activity, according to the classification of Tahir et al.,^45^ agreeing with the porous surface noticed in SEM images and the high presence of V_o_ observed in the Raman and XPS data. Even at higher current densities (Figure 9b), CeO_2_ (RT) obtained from MOF-Ce synthesized at room temperature always showed better electrocatalytic activity compared to CeO_2_ (H).

It is widely known the relationship between the high presence of oxygen defects and OER electrocatalytic activity, affects the absorption of intermediates and consequently the rate-determining step, also modifying the electronic structure and electronic conductivity.^46^ However, although CeO_2_ (H) has a higher *V*_o_ content (48.79%), this did not lead to a lower overpotential compared to the CeO_2_ (RT) sample (*V*_o_ = 43.56%). Miao and co-workers studied the role of oxygen vacancies in OER for PrBaCo_2_O_6−δ_ double perovskite, showing the reduction of the intrinsic OER activity, as the oxygen vacancies largely increase.^47^ The authors demonstrated a change in the structural ordering of the Co^3+^ cation coordination sites, inducing a spin-state transition (from high-spin to low-spin). This change reduces the electronic occupation of the higher energy *e*_g_ orbitals, increasing the electrical resistivity, and decreasing the Co–O bond energy, which is responsible for the reduced OER performance.^47^ Other authors also report the loss of active sites due to the excessive oxygen vacancies in electrocatalysts in the OER.^48,49^ Although in-depth studies have not been conducted for CeO_2_-based materials, the excess oxygen vacancies in this case likely have a negative contribution to electrocatalytic performance, but experiments need to be conducted in the future to investigate this.

The OER kinetics was investigated via Tafel plots (Figures 9c and S6 for pristine Ni foam), obtained by eq 2, where η is the overpotential, a is the intercept relative to the current density (*j*_0_), and *b* is the Tafel slope.^50^ Several kinetic mechanisms were proposed to understand the OER catalytic process,^51^ however, the model proposed by Krasil’shchikov fits well for OER catalyzed by metal oxides in an alkaline medium,^52^ described in the following equation. The calculated values were 58.92 and 62.25 mV dec^–1^ for CeO_2_ (H) and CeO_2_ (RT), respectively. Thus, for both materials, the rate-determining step is the formation of O^–^ species on the electrocatalyst’s surface.
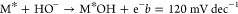
5
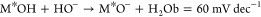
6
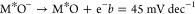
7
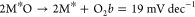
8

The electrical double-layer capacitance (*C*_DL_) values were obtained from the linear relationship between the anode peak current and the scan rate (*i*_a_ vs υ, Figure 9d), measured in cyclic voltammetry experiments (Figure S7). From the *C*_DL_ values, the electrochemically active surface areas (ECSA, Figure 9e) were estimated by eq 4, considering the specific capacitance equal to 0.040 mF cm^–2^.^11^ The CeO_2_ (H) shows a value of *C*_DL_ equal to 12.28 mF and ECSA equal to 307 cm^2^, while the calculated values for CeO_2_ (RT) were *C*_DL_ = 16.92 mF and ECSA = 423 cm^2^. Therefore, the high ECSA value for the CeO_2_ (RT) electrocatalyst agrees with the lower overpotential. Since this electrocatalyst has a higher concentration of Ce^4+^ ions on the surface (87.55%) compared to the CeO_2_ (H) (81.75%), these ions must play an important role in the high catalytic activity. E.g., Chen and co-workers also observed a strong correlation was observed between the OER performance and Ce^4+^ concentration in cerium-modified copper oxide (CuO_*x*_).^53^ As explained previously, the presence of Ce^3+^ contributes to the formation of oxygen vacancies, which may contribute positively to reducing the OER overpotential, as long as the *V*_o_ concentrations are not excessive. However, Yu et al. also demonstrated that the formation of the Ce^4+^/Ce^3+^ redox couple in CeO_2–*x*_ electrocatalysts optimizes oxygen-binding free energies, boosting the OER electrocatalytic activity.^54^ In this way, the Ce^4+^/Ce^3+^ proportion in the CeO_2_ (RT) electrocatalyst should be more suitable, playing a key role in obtaining high ECSA and so lower OER overpotential.

Chronopotentiometry experiments (Figure 9f) demonstrated high stability for up to 24 h for both cerium oxides, with no significant variations in the overpotential. After this, the electrocatalysts were analyzed via SEM and XRD, and the results are shown in Figure S9. A total conservation of the morphology for both electrocatalysts was observed. In addition, the diffraction patterns showed characteristic signals of the substrate (Ni foam) and the cerium oxide, both for CeO_2_ (H) and CeO_2_ (RT), indicating that in addition to the morphology, the crystal structure remains unchanged after the electrocatalytic process.

**Figure 10:**
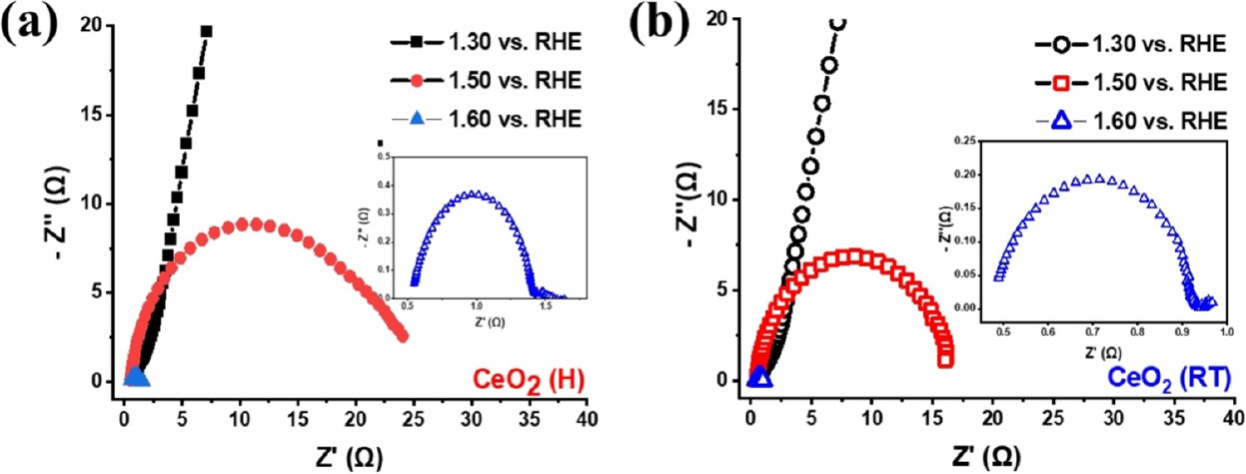
Nyquist plots from the ESI data for (a) CeO_2_ (H) and (b) CeO_2_ (RT) electrocatalysts.

A more detailed investigation of the electrocatalytic activity was carried out using Electrochemical Impedance Spectroscopy, and the results are shown in Figure 10 (Nyquist plots) and Figure S9 (Bode plots). Experiments were conducted using potential before, during, and after OER (1.30, 1.50, and 1.60 V vs RHE). The Bode plots for both electrocatalysts (Figure S9) indicate that the process occurs in a single time constant (τ = *RC*), therefore a more simplified equivalent circuit was adopted (*R*_s_(*R*_CT_*Q*_CPE_), insert in Figure 10),^55^ where *R*_s_ is the electrolyte resistance, *R*_CT_ is the charge transfer resistance and *Q*_CPE_ is a constant phase element. From the *R*_CT_ and QCPE values, the *C*_DL_ was calculated according to the equation (*C*_DL_ = *R*_CT_^(1–*n*)^/*nQ*_CPE_^1/*n*^). Table 3 expresses the results of the EIS adjustments. Experimental values of *R*_CT_ during OER for CeO_2_ (RT) electrocatalyst were 15.67 Ω, lower than the CeO_2_ (H) (20.75 Ω), also agreeing with the observed overpotential since this indicates a faster electron transfer rate. The literature demonstrates an increase in electrical resistance with the induction of more oxygen vacancies,^47,56^ which perfectly justifies the difference between the CeO_2_ (H) and CeO_2_ (RT) electrocatalytic performances. Calculated *C*_DL_ values were 53.3 and 61.5 mF for CeO_2_ (H) and CeO_2_ (RT), respectively, also in concordance with the superior performance observed for the CeO_2_ (RT) electrocatalysts.

**Table 3:** Results Obtained from the ESI Spectra

electrocatalyst	*R*_s_ (Ω)	*R*_CT_ (Ω)	*C*_DL_ (mF)	*n*	*f* (Hz)
CeO_2_ (H)					
1.30	0.47	4.22 × 10^8^	1.76 × 10^8^	0.53	2.14 × 10^15^
1.50	0.53	20.75	53.3	0.90	1.44 × 10^1^
1.60	0.54	0.88	25	0.89	7.21
CeO_2_ (RT)					
1.30	0.40	8.72 × 10^8^	4.73 × 10^8^	0.53	3.86 × 10^16^
1.50	0.47	15.67	61.5	0.92	1.65 × 10^1^
1.60	0.47	0.45	27.6	0.89	12.7

Although many factors can play an important role in the OER electrocatalytic activity (morphology, *R*_CT_, *C*_DL_, oxygen vacancies, and so on), the η at 10 mA cm^–2^ is the main parameter considered to be the benchmark for the comparison between OER electrocatalysts. Table 4 summarizes the η and Tafel slope values for several CeO_2_ electrocatalysts reported in the literature compared to the results obtained in this work. A considerable number of reports focus on the production of composites and/or Ce-doped materials, and few studies focus on the production of pure CeO_2_ electrocatalysts. A wider comparison can be found in some reviews,^13,16,57^ Although CeO_2_ presents promising properties, OER performances for single-phase CeO_2_-based electrocatalysts in the literature are still moderate.^13,16^ The results shown in Table 4 depicts that CeO_2_ (H) and CeO_2_ (RT) electrocatalyst activities are similar or superior compared to most catalysts based solely on CeO_2_, which demonstrates the high quality of the results obtained in this work.

**Table 4:** Overpotential and Tafel Slope Values for the CeO_2_ (H) and CeO_2_ (RT) Compared to Other CeO_2_-Based Electrocatalysts in the Lliterature, and the Benchmarks (IrO_2_ and RuO_2_)

electrocatalyst	η_10_ (mV)	Tafel (mV dec^–1^)	electrolyte	reference
CeO_2_ (H)	326	58.92	1.0 M KOH	this work
CeO_2_ (RT)	319	62.25	1.0 M KOH	this work
CeO_2_/SS	353	99	1.0 M KOH	(57)
CeO_2_/CC	530	76.2	1.0 M KOH	(58)
CeO_2−δ_	630	288	1.0 M KOH	(59)
CeO_2_	628	158.6	1.0 M KOH	(60)
CeO_2_ (HC)	320	61.6	1.0 M KOH	(61)
CeO_2_/C	297	46	1.0 M KOH	(21)
CeO_2_	580	131	1.0 M KOH	(62)
CeO_2_ nanosheets	310	170	1.0 M KOH	(63)
CeO_2_@PIZA/FTO	370	48	1.0 M KOH	(64)
IrO_2_	351	114	1.0 M KOH	(65)
RuO_2_	326	145	1.0 M KOH	(66)

## 4 Conclusions

Here, two cerium oxides were successfully obtained using MOF-Ce polymorphs as templates with total morphology conservation as observed in the SEM images. CeO_2_ (H) with block-like morphology and CeO_2_ (RT) with rod-like morphology crystallize in fluorite structure, and FT-IR data indicates the presence of oxygenated organic groups in the oxide surfaces. Raman and XPS high-resolution spectra indicate the presence of a high content of oxygen vacancies due to the presence of Ce^3+^ ions in the solid structure. Both electrocatalysts show excellent OER activity with reduced overpotential, hydroxyl ions adsorption as the rate-determining reaction step and stability for up to 24 h. Electrochemical investigations show CeO_2_ (RT) superior electrocatalytic performance due to the suitable presence of oxygen vacancies, leading to low charge-transfer resistance, high *C*_DL_ value, and large ECSA.

## Supporting Information Available

Additional information (FT-IR, XPS, LSV, CV, SEM, TEM, XRD, and EIS data) (PDF)
