# Supplementary material for: Impact of the Metal–Organic Frameworks Polymorphism on the Electrocatalytic Properties of CeO2 toward Oxygen Evolution
Source: ACS Omega. 2024 Nov 29;9(50):49913–24. doi: 10.1021/acsomega.4c08837 (PMC11656357; doi:10.1021/acsomega.4c08837)
Supplement: Supplementary file 1 — ao4c08837_si_001.pdf [file ao4c08837_si_001.pdf]

Impact of the Metal-Organic Frameworks Polymorphism on the Electrocatalytic Properties of CeO_2 Toward Oxygen Evolution

Nicolle Pauline de Araújo Mendes[a], Antonio Lopes de Souto Neto [a], Johnnys da Silva Hortêncio[a], André L. Menezes de Oliveira[b], Rafael A. Raimundo[c,d], Daniel Araújo Macedo[d], Fausthon Fred da Silva,[a]*.

[a] *Departamento de Química, Universidade Federal da Paraíba (UFPB), 58.051-900, João Pessoa – PB, Brazil.*

[b] *Núcleo de Pesquisa e Extensão LACOM, Departamento de Química, Universidade Federal da Paraíba, 52051-85, João Pessoa-PB, Brazil*

[c] *TEMA - Centre for Mechanical Technology and Automation, Department of Mechanical Engineering, University of Aveiro, 3810-193, Aveiro, Portugal.*

[d] *Programa de Pós-Graduação em Ciência e Engenharia de Materiais - PPCEM, Universidade Federal da Paraíba (UFPB), 58.051-900, João Pessoa, PB, Brazil.*

*Corresponding author: fausthon@quimica.ufpb.br

Supplementary information file

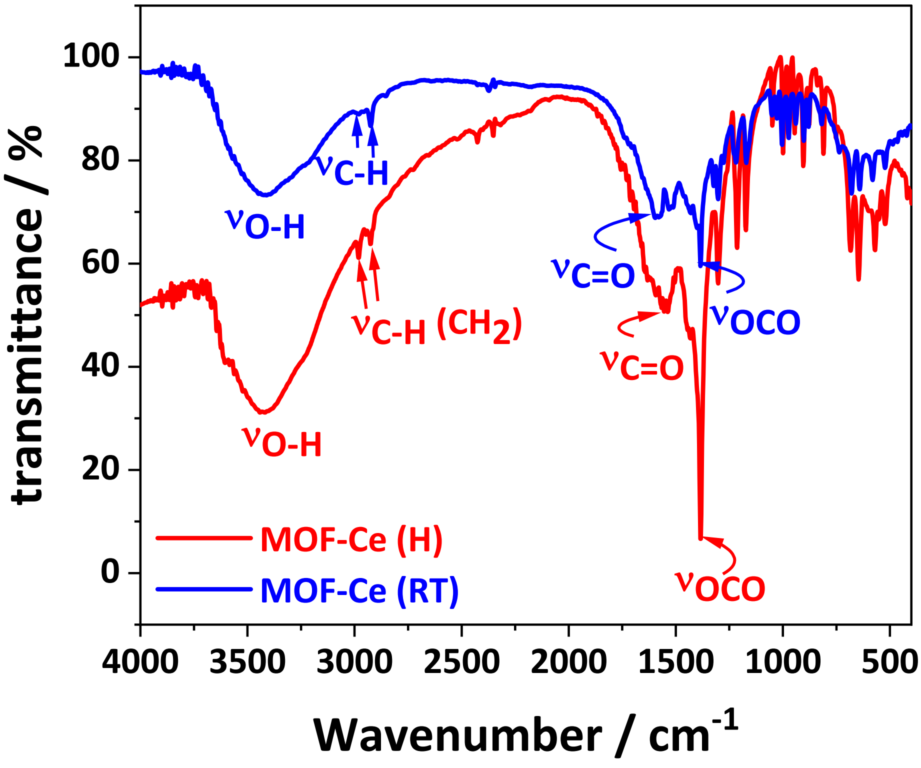

Figure S1: MOF-Ce (H) and MOF-Ce (RT) infrared spectra.

Table S1: Rietveld refinement data for the CeO_2 samples.

samples	*CeO_2 – ICSD 61595*		*agreement factors*		
	Size (nm)	a (Å)	R_{wp} (%)	R_{exp} (%)	χ^2
CeO_2 – ICSD 61595	---	5.41	---	---	---
Sample 1	8.3	5.41	9.41	10.06	**0.94**
Sample 2	6.2	5.41	9.49	10	**0.95**

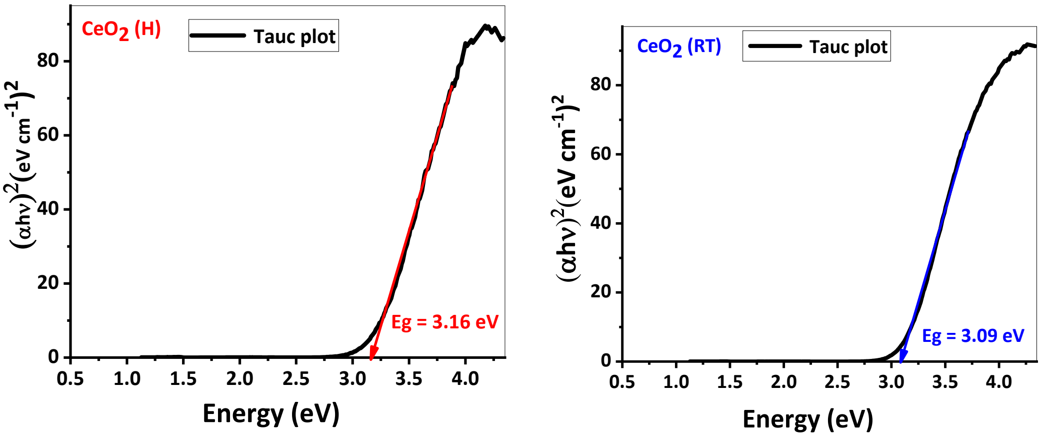

Figure S2: Tauc plots and bandgaps of the CeO_2 (H) and CeO_2 (RT).

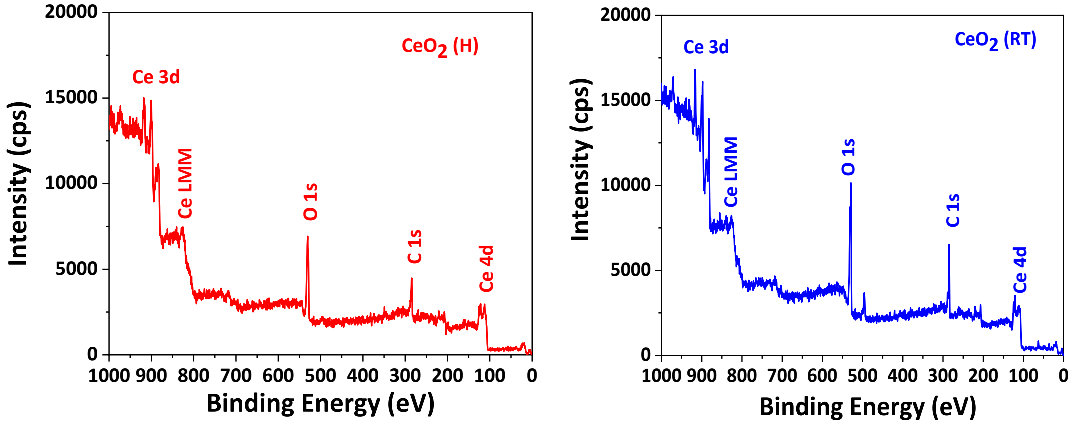

Figure S3: XPS survey spectra of CeO_2 (H) and CeO_2 (RT)

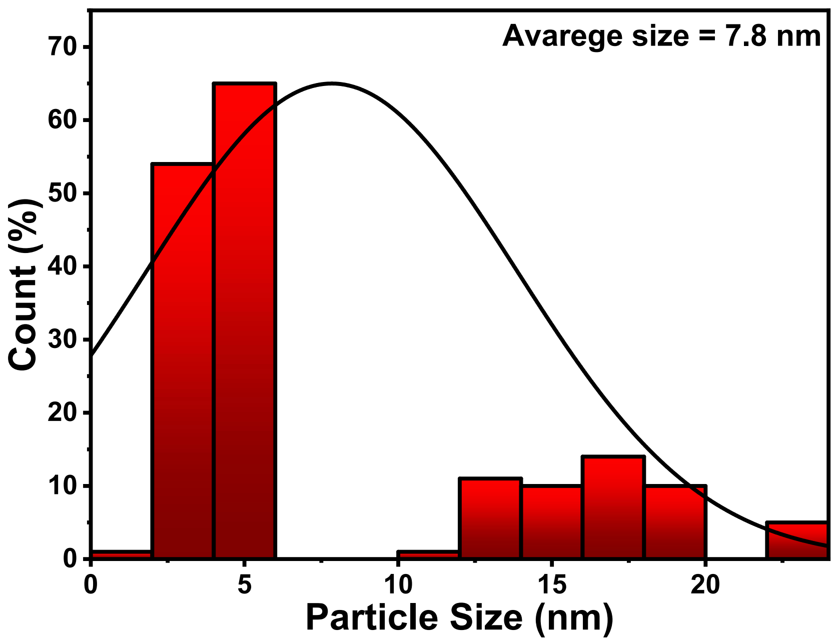

Figure S4: Particle size distribution for the CeO_2 (H) sample.

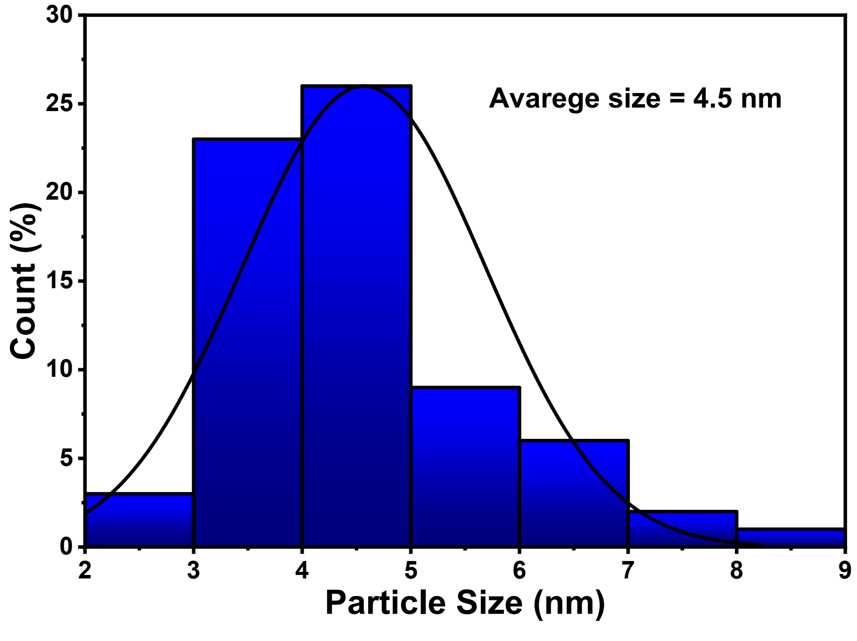

Figure S5: Particle size distribution for the CeO_2 (RT) sample.

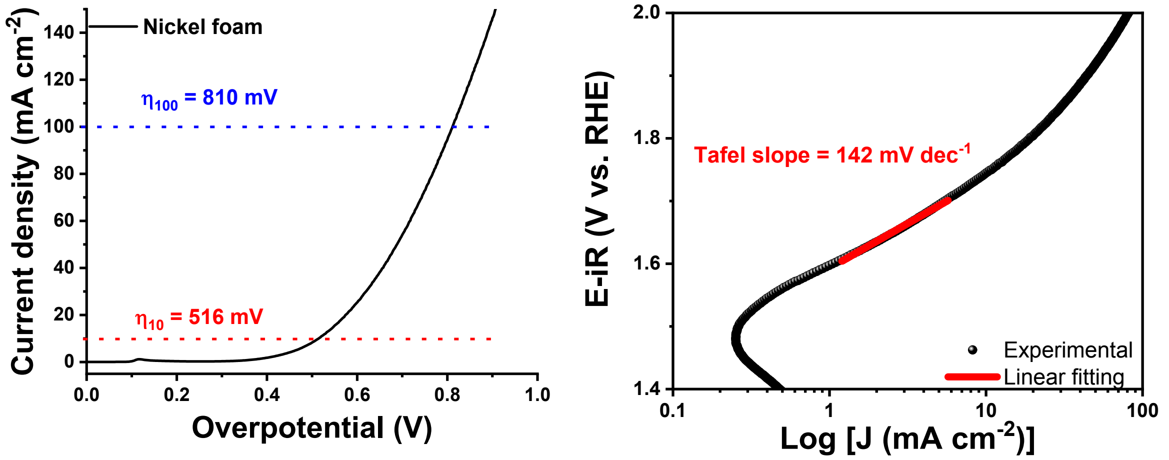

Figure S6: LSV curve and Tafel slope of the Ni foam substrate.

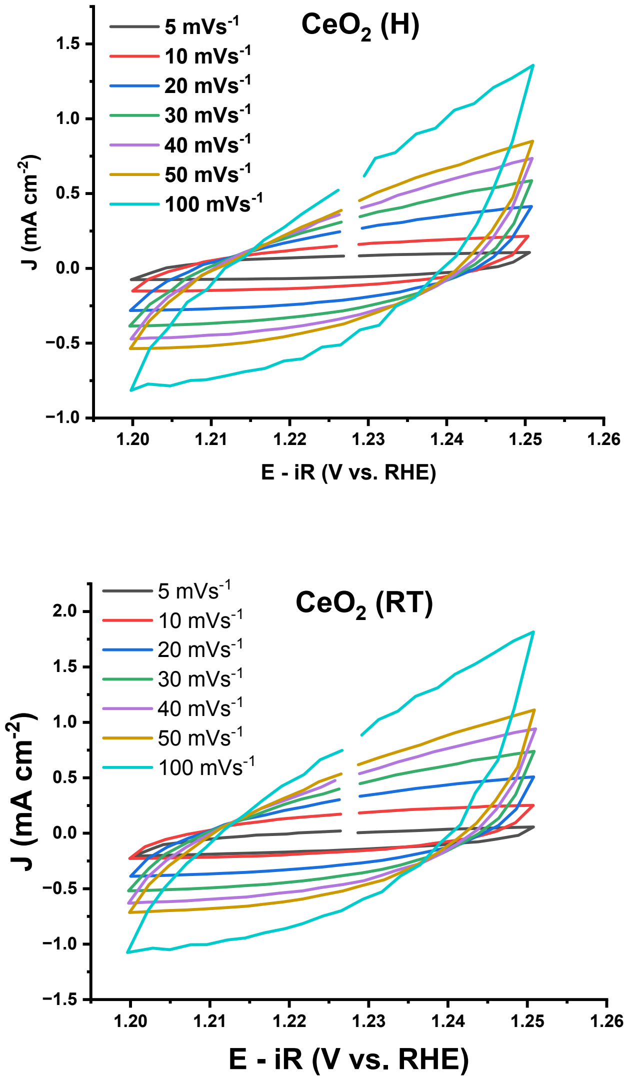

Figure S7: Cyclic Voltammograms of CeO_2 (H) and CeO_2 (RT) with scanning range of 10-100mV.

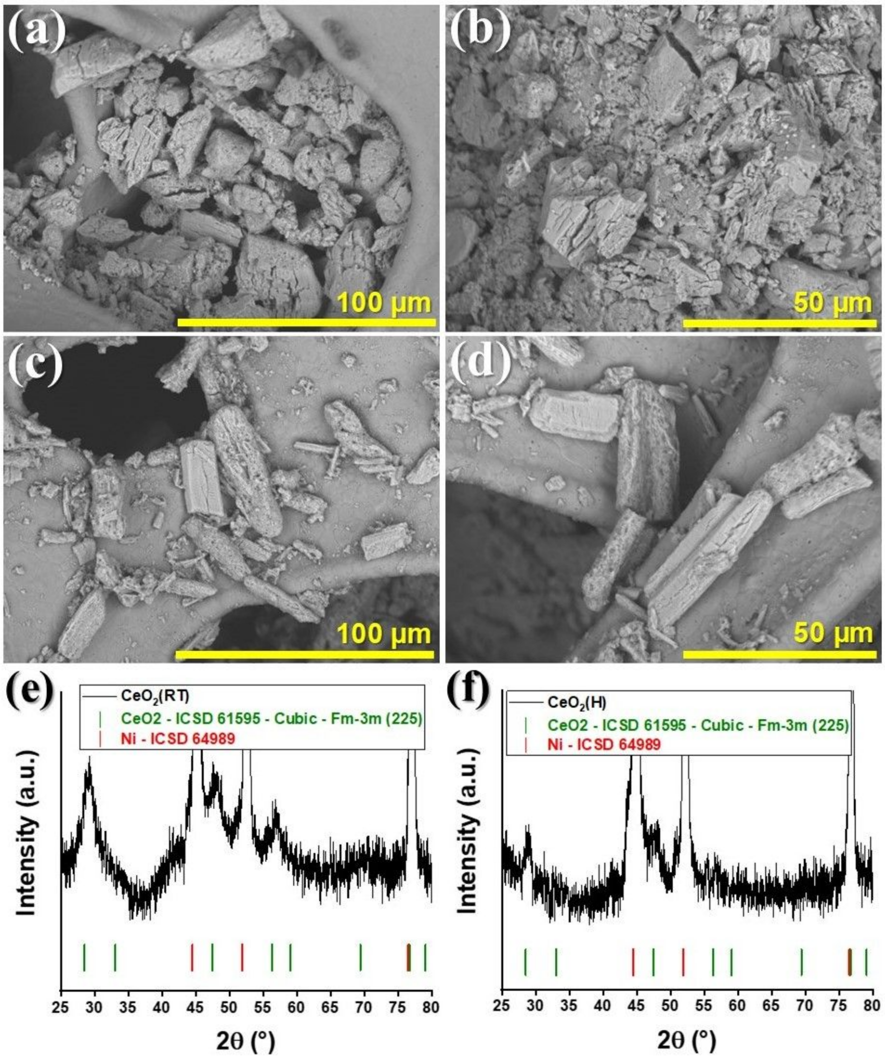

Figure S8: SEM images and XRD data for CeO_2 (H) (a,b,f) and CeO_2 (H) (c,d,e) electrocatalysts after the OER electrocatalysis

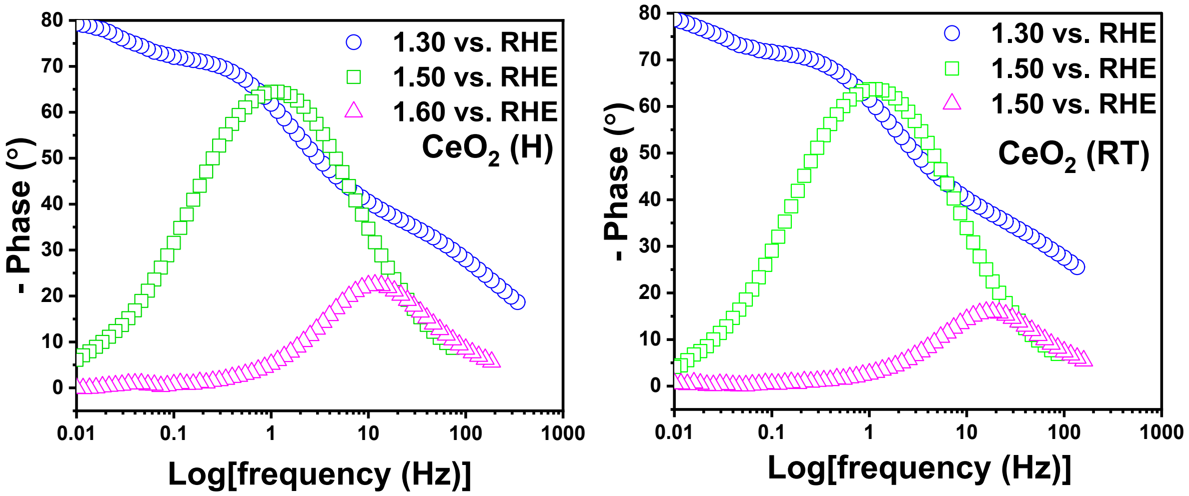

Figure S9: Bode plots of the electrocatalysts.
